# Secondary predation constrains DNA-based diet reconstruction in two threatened shark species

**DOI:** 10.1038/s41598-021-96856-w

**Published:** 2021-09-15

**Authors:** Mark de Bruyn, Matteo Barbato, Joseph D. DiBattista, Matt K. Broadhurst

**Affiliations:** 1 School of Life and Environmental Sciences, The University of Sydney, Sydney, NSW 2006 Australia; 2 Department of Biology, University of Padova, Padova, Italy; 3 Australian Museum Research Institute, Australian Museum, 1 William Street, Sydney, NSW 2010 Australia; 4 NSW Department of Primary Industries, Fisheries Conservation Technology Unit, National Marine Science Centre, Southern Cross University, 2 Bay Drive, Coffs Harbour, NSW 2450 Australia; 5 Marine and Estuarine Ecology Unit, School of Biological Sciences, University of Queensland, St Lucia, Australia

**Keywords:** High-throughput screening, Ecological genetics

## Abstract

Increasing fishing effort, including bycatch and discard practices, are impacting marine biodiversity, particularly among slow-to-reproduce taxa such as elasmobranchs, and specifically sharks. While some fisheries involving sharks are sustainably managed, collateral mortalities continue, contributing towards > 35% of species being threatened with extinction. To effectively manage shark stocks, life-history information, including resource use and feeding ecologies is pivotal, especially among those species with wide-ranging distributions. Two cosmopolitan sharks bycaught off eastern Australia are the common blacktip shark (*Carcharhinus limbatus*; globally classified as Near Threatened) and great hammerhead (*Sphyrna mokarran*; Critically Endangered). We opportunistically sampled the digestive tracts of these two species (and also any whole prey; termed the ‘Russian-doll’ approach), caught in bather-protection gillnets off northern New South Wales, to investigate the capacity for DNA metabarcoding to simultaneously determine predator and prey regional feeding ecologies. While sample sizes were small, *S. mokkaran* fed predominantly on stingrays and skates (Myliobatiformes and Rajiformes), but also teleosts, while *C. limbatus* mostly consumed teleosts. Metabarcoding assays showed extensive intermixing of taxa from the digestive tracts of predators and their whole prey, likely via the predator’s stomach chyme, negating the opportunity to distinguish between primary and secondary predation. This Russian-doll effect requires further investigation in DNA metabarcoding studies focussing on dietary preferences and implies that any outcomes will need to be interpreted concomitant with traditional visual approaches.

Global biodiversity is under threat, with accelerating losses of species and increasing concern about ecosystem changes^1^. In the oceans, escalating fishing effort and associated bycatches and discarding practices are impacting on marine biodiversity^2^. Levels of extinction threat vary regionally and by taxonomic group, but owing to their low reproductive rates and late age-at-maturity, elasmobranchs, especially sharks, are among those taxa highly susceptible to increasing anthropogenic pressures^3^. More specifically, among the total known species of ‘ground sharks’ or the Carcharhiniformes (*n* = 287), 36% are listed as threatened with extinction by the International Union for Conservation of Nature (IUCN), and a further 18% are classified as ‘Data Deficient’^4^. To mitigate this extinction threat, there is a critical need for more information on the ecology of sharks, particularly those that are threatened or endangered.

In Australia, several shark species are targeted in commercial gillnet fisheries (e.g. gummy sharks *Mustelus antarcticus* and sandbar sharks *Carcharhinus plumbeus*^5,6^; and also in bather-protection programs using gillnets and/or baited hooks (e.g. white *Carcharodon carcharias* tiger *Galeocerdo cuvier*, and bull sharks *Carcharhinus leucas*^7,8^). Various ancillary species also incur collateral mortalities as bycatch from the above and other commercial fishing^9–11^.

Two of the more abundant, cosmopolitan Carcharhiniformes found in Australia and well represented in bycatches of bather-protection fishing gears^7,8^ are the common blacktip shark (*Carcharhinus limbatus*) and great hammerhead (*Sphyrna mokarran*), which are globally classified as Near Threatened and Endangered, respectively^4^. *Sphyrna mokarran* is also registered in Appendix II of the Convention on International Trade in Endangered Species (www.cites.org), which has precipitated their legislated protection across many jurisdictions. Notwithstanding these classifications, the biology and ecology of *S. mokarran* remain poorly understood, particularly their diet, which limits effectively managing their remaining stocks^3,12,13^.

*Sphyrna mokarran* can reach a maximum size of 6.0 m total length (TL) and in situ observations imply it predominantly feeds on rays^14–16^ but, like most of its apex congeners also consumes teleosts and other sharks ^12,13,17,18^. Nevertheless, data are limited and in a recent review Gallagher and Klimley^19^ stated that *S. mokarran* feeding ecology requires further assessment. Relatively more is known about the smaller, mesopredator *C. limbatus* (maximum TL of ~ 2.6 TL), which typically feeds on teleosts throughout their cosmopolitan distribution^20–22^. Nevertheless, much of the research describing *C. limbatus* is restricted to the Atlantic Ocean and less is known about their foraging ecology off Australia.

Shark feeding ecology has been quantified mainly by visually identifying stomach contents, biochemical techniques (e.g. stable isotope, lipid and amino acid signatures) or telemetry (e.g. satellite and acoustic tagging) via habitat association^23^. Each of these methods has inherent strengths and weaknesses (reviewed in^24,25^). More recent innovations in genetic techniques, particularly high-throughput metabarcoding approaches, are now routinely contributing to our understanding of predator–prey relationships, and in general, provide improved taxonomic resolution of prey vs. traditional methods^26–28^. The DNA metabarcoding of digestive-tract contents is now commonplace in studies on terrestrial taxa (reviewed in^26,29,30^) and recently has also been applied to marine taxa, mostly teleosts and elasmobranchs (e.g.^31–37^).

A promising application of DNA metabarcoding for digestive tract content analyses is in the reconstruction of trophic food webs^27^. Where prey items remain relatively intact in a predator’s digestive tract, it should be possible to sequence the digestive-tract contents of both the predator and its prey to determine multiple levels of trophic interactions^27,38^. However, one aspect of this so-called ‘Russian-doll’ approach^39^ that deserves further attention is the extent to which DNA intermixing occurs between predator and (whole) prey digestive tracts, via the predator’s stomach chyme. Considering the above, our objectives here were to use opportunistically sampled *S. mokarran* and *C. limbatus* caught in bather-protection gillnets off eastern Australia to further investigate DNA metabarcoding to trace 'Russian-doll’ trophic interactions in marine predators.

## Material and methods

### Ethics declaration

All experimental protocols were approved by the NSW Department of Primary Industries (Australian government), and carried out in accordance with relevant guidelines and regulations. All methods reported are in accordance with ARRIVE guidelines (https://arriveguidelines.org).

### Sample collection

**Figure 1:**
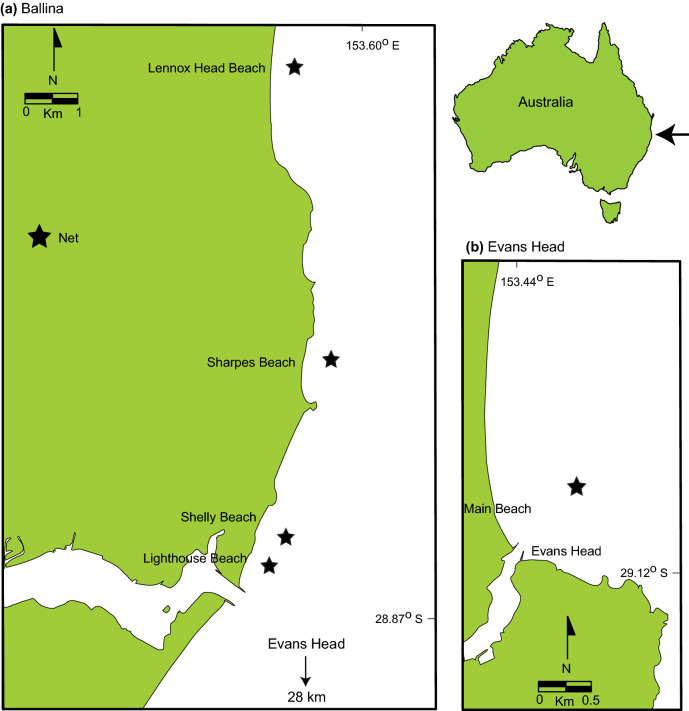
Location of bather-protection gillnets deployed off (**a**) Ballina and (**b**) Evans Head in NSW, Australia from which sharks were sampled between 7 February and 13 March, 2018. Maps were taken and modified from www.outline-worldmap.com.

The study was done using seven *S. mokarran* (three females and four males) and four *C. limbatus* (two of each sex) that died in bather-protection gillnets deployed off Lennox Head, Ballina, and Evans Head, NSW, Australia (28.77° S, 153.60° E to 29.10° S, 153.44° E) between 7 February and 13 March, 2018 (Fig. 1). Each gillnet measured 150 m long × 4 or 6 m deep and comprised 600- or 800-mm mesh made from either 1.8- or 2.1-mm diameter twisted polyethylene, or 2.5-mm diameter polyamide twine (see^8^ for details of the fishing gear).

Whole sharks were removed from the gillnets and stored at  − 20 °C (within 3 h) until necropsied in May 2018. During the necropsy process, specimens were defrosted for 12 h and measured for TL before the stomach cavity was opened in a sterile field laboratory with bleach sterilized tools. All digestive contents were removed, with any animal matter identified (where possible), preserved in 100% ethanol, and stored at  − 20 °C until further analyses. Extraction controls for DNA (ultrapure water samples, Invitrogen, Waltham, USA) were collected in sterile 1.5 mL microcentrifuge tubes alongside the stomach-content samples and subjected to the same workflow described below.

### Stomach-content analyses and DNA extraction

**Figure 2:**
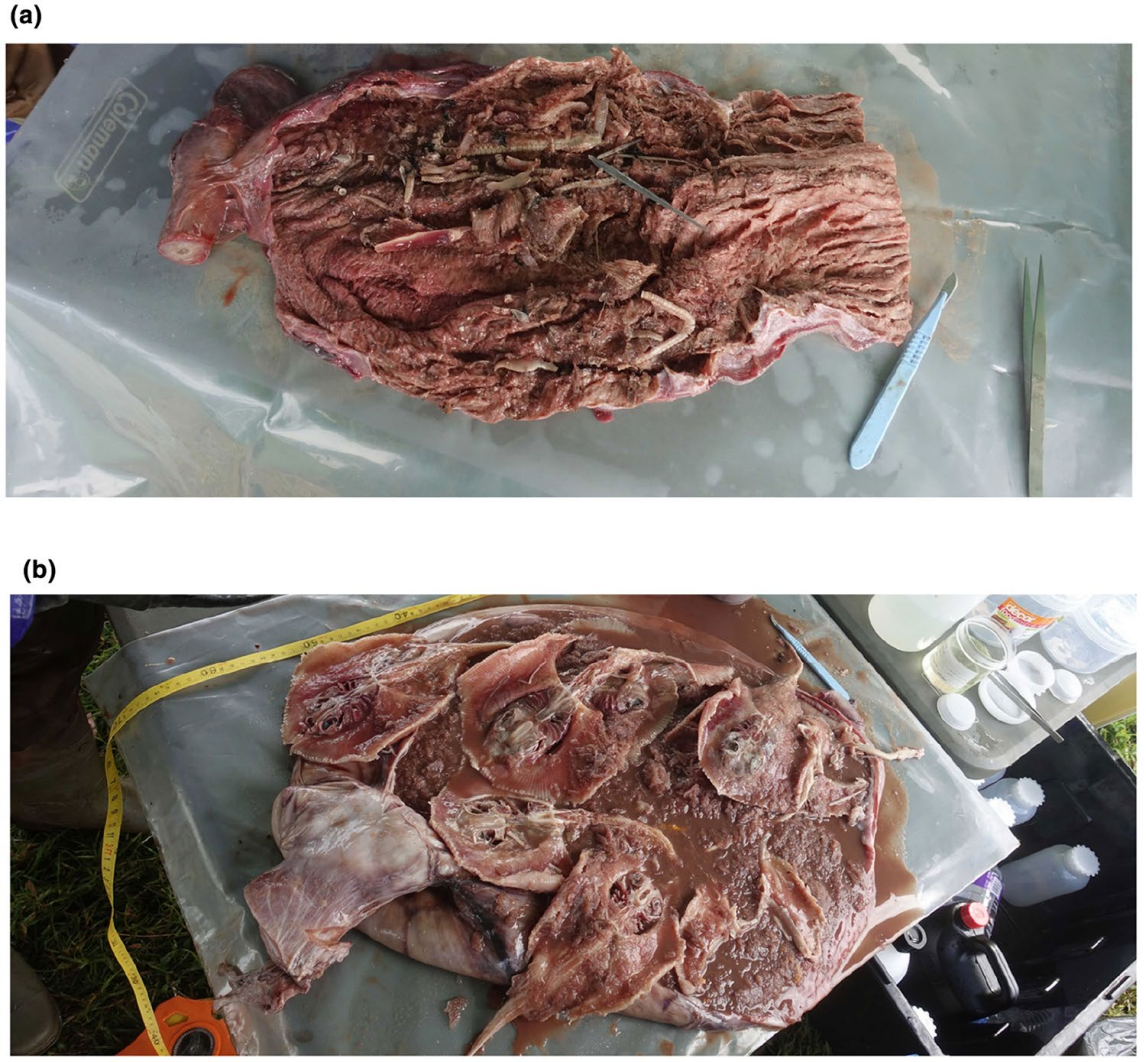
Photos of (**a**) jaw bones and (**b**) *Urolophus* sp. in the stomachs of two *Sphyrna mokarran.*

Whole prey removed from *S. mokkaran* and *C. limbatus* were identified by visual inspection, most to the species level. Stomach contents of some of these whole prey items, comprising seven individual *Urolophus* sp. and two *A. rostrata* rays, predated by three *S. mokarran* individuals, were included in the metabarcoding analysis (Table 1). Some of the incomplete specimens, typically teleost jawbones or scales, were identified to genus only (Fig. 2). For each *S. mokkaran* and *C. limbatus*, and each whole prey item detailed above, the remainder of its unidentifiable stomach contents (both liquids and solids) were separately homogenised using a previously sterilised (washed with detergent, followed by a 10% bleach wash, and rinsed thoroughly in MilliQ water) commercial food blender.

**Table 1:** Sample identification, species and sex (where identifiable) and the contents of their stomachs (‘gut’) determined via metabarcoding (to the closest taxonomic level) and through visual inspection.

Sample ID	Species (sex)	Gut 12S reads: ≥ 95% query coverage, ≥ 90% identity match	Gut 12S reads: 100% query coverage, ≥ 97% identity match	Gut 16S reads ≥ 95% query coverage, ≥ 90% identity match	Gut 16S reads: 100% query coverage, ≥ 97% identity match	Gut 18S reads: ≥ 95% query coverage, ≥ 90% identity match	Gut 18S reads: 100% query coverage, ≥ 97% identity match	Stomach contents weight (g)	Whole or partial prey items in gut (sample ID in bold if sequenced)
1H	*S. mokarran* (M)	n/a	n/a	*Portunus* spp. (142,213)	*Portunus* spp. (142,213)	Carcharhinidae (1977); Chondrichthyes (4662)	Carcharhinidae (2364); Chondrichthyes (3444); Myliobatiformes (74)	100	0
2H	*S. mokarran* (F)	n/a	n/a	*Sphyrna mokarran* (19)	*Sphyrna mokarran* (19)	Carcharhinidae (4354); Chondrichthyes (8)	Carcharhinidae (4331); Chondrichthyes (2)	789	
3H	*S. mokarran* (F)	Carcharhinidae (76,525)	Carcharhinidae (76,525)	*Callianassa *spp. (140)	*Callianassa *spp. (140)	Actinopteri (1); Carcharhinidae (11,656); Chondrichthyes (2738)	Carcharhinidae (12,456); Chondrichthyes (1503); Myliobatiformes (4)	6264	9 whole *Urolopus* sp. (3A-3D)
4H	*S. mokarran* (F)	*Gnathophis* spp. (332); *Trachurus* spp. (231); *Lepidotrigla* spp. (5607); *Pseudorhombus* spp. (10); *Anoplocapros* spp. (61,831); Carcharhinidae (2555)	*Gnathophis* spp. (332); *Trachurus* spp. (231); *Lepidotrigla* spp. (5607); *Anoplocapros* spp. (61,831); Carcharhinidae (2,555)	*Portunus* spp. (18,030); *Ranina *spp. (36,346)*; Penaeoidea* spp. (1347); *Sphyrna mokarran* (2)	*Portunus* spp. (18,030); *Penaeoidea *spp. (1347); *Sphyrna mokarran* (2)	Cichlidae (17); Actinopteri (46); Carcharhinidae (7230); Chondrichthyes (5179)	Anguilliformes (3); Cichlidae (10); Actinopteri (21); Carcharhinidae (7935); Chondrichthyes (3945)	6149	3 *Aptychotrema rostrata* (4SN, 4SN2); 3 *Urolopus* sp. (4SR, 4*)
5H	*S. mokarran* (M)	*Platycephalus* spp. (24,007); *Dexillus* spp. (35,992); *Anoplocapros* spp. (57,956); Carcharhinidae (4)	*Anoplocapros* spp. (57,956); Carcharhinidae (4)	*Callianassa* spp. (365)*; Trachysalambria* spp. (87,918)	*Callianassa* spp. (365)	Cichlidae (470); Actinopteri (611); Carcharhinidae (4228); Chondrichthyes (5200)	Anguilliformes (21); Cichlidae (447); Actinopteri (314); Carcharhinidae (4798); Chondrichthyes (4350); Myliobatiformes (2)	3319	*Lactoria fornasini*; 3 + *Urolopus* sp.; 2 *Sillago* sp.; *Anoplocapros inermis* (2 scales)
6H	*S. mokarran* (M)	*Dexillus* spp. (506); *Anoplocapros* spp. (1428); Carcharhinidae (63,304)	*Anoplocapros* spp. (1428); Carcharhinidae (63,304)	*Sphyrna mokarran* (103)	*Sphyrna mokarran* (103)	Carcharhinidae (5619); Chondrichthyes (5200)	Carcharhinidae (5541)	384	0
7H	*S. mokarran* (M)	*Gnathanacanthus* spp. (57,053); *Anoplocapros* spp. (2318); Diodontidae (35,675); *Monocentris* spp. (2740); Carcharhinidae (4016)	*Anoplocapros* spp. (2318); Diodontidae (35,675); *Monocentris* spp. (2740); Carcharhinidae (4016)	0	0	Cichlidae (10); Actinopteri (36); Carcharhinidae (4188); Chondrichthyes (402)	Actinopteri (29); Carcharhinidae (3559); Chondrichthyes (239); Myliobatiformes (12)	5300	1 *Helicolenus percoides*.; 1 *Urolopus* sp. (7SR); 1 + *Dicotylichthys punctulatus* (jawbone)
CH	Sample control – *S. mokarran*	0	0	0	0	0	0	n/a	n/a
PCRneg1	PCR negative 1	0	0	0	0	*Homo sapiens* (8126)	*Homo sapiens* (8126)	n/a	n/a
1BT	*C. limbatus* (M)	*Hyperlophus vittatus* (19,547); *Gerres* spp. (25,466); *Dexillus* spp. (378); *Anoplocapros* spp. (933); Carcharhinidae (72);	*Hyperlophus vittatus* (19,547); *Anoplocapros* spp. (933); Carcharhinidae (72)	Carcharhinidae (16)	Carcharhinidae (16)	Cichlidae (19); Actinopteri (6); Carcharhinidae (1739); Chondrichthyes (5)	Actinopteri (25); Carcharhinidae (1); Chondrichthyes (4)	708	0
2BT	*C. limbatus* (F)	Carcharhinidae (63,975)	Carcharhinidae (63,975)	*Chrysomya* spp. (373); Carcharhinidae (30)	*Chrysomya* spp. (373); Carcharhinidae (30)	Carcharhinidae (5223)	0	232	0
3BT	*C. limbatus* (M)	Carcharhinidae (10,460)	Carcharhinidae (10,460)	0	0	Carcharhinidae (6201); Chondrichthyes (9)	Carcharhinidae (591); Chondrichthyes (1)	195	0
4BT	*C. limbatus* (F)	*Platycephalus* spp. (1636); *Dexillus* spp. (2011); *Anoplocapros* spp. (4461); Carcharhinidae (24,679)	*Anoplocapros* spp. (4461); Carcharhinidae (24,679)	*Lucilia* spp. (2)	*Lucilia* spp. (2)	Carcharhinidae (4008); Chondrichthyes (9)	0	297	0
CBT	Sample Control – *C. limbatus*	Carcharhinidae (1145)	Carcharhinidae (1145)	0	0	*Homo sapiens* (575)	*Homo sapiens* (575)	n/a	n/a
PCRneg2	PCR negative 2	0	0	0	0	*Homo sapiens* (1679)	*Homo sapiens* (1679)	n/a	n/a
3A	*Urolopus* sp.	*Homo sapiens* (3); Carcharhinidae (4643)	*Homo sapiens* (3); Carcharhinidae (4643)	0	0	Chondrichthyes (9489)	Carcharhinidae (11); Chondrichthyes (9476)	unknown	0
3B	*Urolopus* sp.	Carcharhinidae (18,590)	Carcharhinidae (18,590)	*Callianassa* spp. (143,578); *Matuta* spp. (71)	*Callianassa* spp. (143,578)	Callianassidae (10); Actinopteri (1); Carcharhinidae (43); Chondrichthyes (7934)	Callianassidae (10); Carcharhinidae (128); Chondrichthyes (7857)	unknown	0
3C	*Urolopus* sp.	Carcharhinidae (5135)	Carcharhinidae (5135)	*Callianassa* spp. (66,115)	*Callianassa* spp. (66,115)	0	0	unknown	0
3D	*Urolopus* sp.	Carcharhinidae (3923)	Carcharhinidae (3923)	0	0	Actinopteri (3); Carcharhinidae (3208); Chondrichthyes (14,433)	Carcharhinidae (40); Chondrichthyes (14,403)	unknown	0
4SR	*Urolopus* sp.	*Gnathophis* spp. (69); *Lepidotrigla* spp. (958); *Pseudorhombus* spp. (50); *Anoplocapros* spp. (29,358); Rhinobatidae (2); Carcharhinidae (41,434)	*Gnathophis* spp. (69); *Lepidotrigla* spp. (958); *Anoplocapros* spp. (29,358); Carcharhinidae (41,434)	*Sphyrna mokarran* (2)	*Sphyrna mokarran* (2)	Actinopteri (9); Carcharhinidae (3208); Chondrichthyes (9806)	Carcharhinidae (5506); Chondrichthyes (7071)	unknown	0
4SN	*Aptychotrema rostrata*	*Lepidotrigla* spp. (11); *Pseudorhombus* spp. (57,577); *Anoplocapros* spp. (6329); Rhinobatidae (9815); Carcharhinidae (2335)	*Lepidotrigla* spp. (11); *Anoplocapros* spp. (6329); Carcharhinidae (2335)	*Portunus* spp. (67,051); *Ranina* spp. (18,815)	*Portunus* spp. (67,051)	Decapoda (3); Actinopteri (3); Carcharhinidae (74); Chondrichthyes (16,656)	Decapoda (3); Actinopteri (3); Carcharhinidae (230); Chondrichthyes (16,462)	unknown	0
4SN2	*Aptychotrema rostrata*	*Lepidotrigla* spp. (4); *Anoplocapros* spp. (1190)	*Lepidotrigla* spp. (4); *Anoplocapros* spp. (1190)	n/a	n/a	Actinopteri (7360); Carcharhinidae (533); Chondrichthyes (6474)	Actinopteri (7176); Carcharhinidae (625); Chondrichthyes (6465)	unknown	0
4*	*Urolopus* sp.	*Anoplocapros* spp. (51); Rhinobatidae (2); Carcharhinidae (2)	*Anoplocapros* spp. (51); Carcharhinidae (2)	*Callianassa* spp. (1); *Penaeoidea* spp. (68,616)	*Callianassa* spp. (1); *Penaeoidea* spp. (68,616)	Actinopteri (271); Carcharhinidae (1); Chondrichthyes (13,045)	Actinopteri (271); Carcharhinidae (35); Chondrichthyes (13,020)	unknown	0
7SR	*Urolopus* sp.	*Gnathanacanthus* spp. (22,770); Carcharhinidae (84)	Carcharhinidae (84)	*Callianassa* spp. (25,765); *Portunus* spp. (273)	*Callianassa* spp. (25,765); *Portunus* spp. (273)	Callianassidae (29); Decapoda (81); Actinopteri (48); Carcharhinidae (43); Chondrichthyes (6888)	Callianassidae (29); Decapoda (81); Carcharhinidae (193); Chondrichthyes (6726)	unknown	0

Prey items identified by metabarcoding assays are listed for each of two filtering stringencies per amplicon (see Methods). Values in parentheses after species designation show the number of reads observed for that taxon (values listed in red reflect ≤ 10 reads; ‘n/a’ indicates that the sample was not sequenced, while ‘0’ indicates no amplification of relevant taxa). Species IDs shown in bold are those taxa identified in both the predator and the prey’s digestive tract via metabarcoding, and likely reflect cross-contamination via the predator’s stomach ‘chyme’.

For all samples, DNA was extracted from approximately 5 mL of the homogenate using a QIAamp PowerFecal DNA kit (Qiagen, Sydney, Australia) according to manufacturer’s instructions. This kit effectively removes PCR inhibitors from fecal and stomach content samples. The DNA extraction was carried out in a pre-PCR laboratory to minimise contamination, and clean-room protocols were followed with extensive bleaching and UV treatment of the area and equipment for all laboratory steps. Filter pipette tips were used in all instances and gloves were frequently changed, particularly between handling specimens or plasticware.

### PCR amplification and Illumina sequencing

The DNA extracts from each stomach sample were amplified, tagged separately, and then pooled for sequencing. Two group-specific mini-barcode primers were selected for the amplification of teleost and crustacean DNA, targeting 12S (MiFish^40^) and 16S (Crust16S short^41^) mitochondrial DNA genes, respectively. We also used a universal 18S primer set^42^ targeting the hypervariable V4 region of the nuclear small subunit ribosomal DNA to amplify templates from a broader fraction of marine metazoans. Polymerase chain reaction (PCR) was performed using the AmpliTaq Gold 360 protocol and thermocycling conditions recommended in^30^.

The PCR hybridization temperatures were 50, 51 and 50 °C for MiFish, Crust16S, and Uni18S primer sets, respectively, and products were run on a 1% agarose gel to confirm amplification of the correct target size (MiFish =  ± 170 bp; Crust16S =  ± 170 bp, Uni18S =  ± 220 bp). A second round of PCR was undertaken with the cleaned PCR products using unique dual-indexed primers for each sample, which included the Illumina-specific sequencing adaptors. PCR products were sent to the Ramaciotti Centre for Genomics at the University of NSW for cleaning, normalising, and pooling prior to paired-end sequencing, which was performed using a 500 cycle MiSeq V3 Reagent Kit on an Illumina MiSeq platform (Illumina, San Diego, CA, USA). Sample demultiplexing based on the incorporated indexes was conducted by the sequencing centre.

### Bioinformatic pipeline

All sequence data were quality filtered prior to taxonomic assignment using the following tools: 1) Geneious v10.1.3 (https://www.geneious.com) for stitching R1 and R2 reads using default settings, trimming low quality reads from the 5'/3' end (quality score = 30), removing adapters, and filtering out reads below a minimum threshold length (150 bp for MiFish, 80 bp for Crust16S, and 250 bp for Uni18S), 2) USEARCH (v11.0.667^43^) for renaming files and file format conversion (.fastq to .fasta), dereplication (identifying unique sequences), removing singletons, removing chimeric sequences, and generating a zero-radius operational taxonomic unit) (ZOTU) table with the UNOISE algorithm.

The ZOTUs were queried against the National Centre for Biotechnology Information’s (NCBI) GenBank nucleotide database (accessed in 2020) using BLASTn with the following settings: percentage identity = 97, query coverage = 100, best hit score edge of 0.05, best hit overhang of 0.25, and an E-value of 1e-3. An additional, less-stringent data set was generated for each assay using the following BLASTn settings: percentage identity = 90, query coverage = 95, best hit score edge of 0.05, best hit overhang of 0.25, and an E-value of 1e-3. The LULU algorithm^44^ was then run to curate the assignments assessing sequence similarity and their co-occurrence patterns with the default parameters: minimum_ratio_type = min, minimum_ratio = 1, minimum_match = 84, minimum_relative_cooccurence = 0.95. This entire process was completed on the Zeus SGI cluster based at the Pawsey Supercomputing Centre in Kensington, Western Australia using an abridged version of the fully automated ‘eDNAFlow' pipeline^45^. All raw sequencing data needed to replicate the study are available from Dryad Digital Repository: https://doi.org/10.5061/dryad.kd51c5b4s.

### Statistical analysis of the Russian-doll effect

To test for the Russian-doll effect, and more specifically, to confirm no significant differences between metabarcoding reads from predators’ stomachs (*S. mokarran*) and those of their whole prey (*Urolophus* sp.; *A. rostrata*), Kruskal’s non-metric multidimensional scaling (NMDS) was applied to each metabarcoding assay (12S, 16S, 18S). These analyses were done using the isoMDS function in the Mass package^46^ in R (R Core Team, 2017)^47^. For comparison, NMDS with stable solution from random starts was implemented, using the metaMDS function in the Vegan R package^48^.

Permutational multivariate analysis of variance (PERMANOVA) was used to assess if the metabarcoding reads from the predator and its whole prey were equivalent, using the ADONIS function in Vegan^48^. Where the null hypothesis was rejected, a similarity percentages breakdown (SIMPER) was applied (using Vegan^48^) to determine the contribution of each dietary species to the dissimilarity between metabarcoding reads from predator and prey stomach contents. For all of these analyses, we tested for differences between predator and whole prey using the number of reads for each taxon identified per assay, and also presence/absence of that taxon per assay.

## Results

Four of the *S. mokarran* had identifiable whole or partial prey in their stomachs (Table 1, Supplementary Table 1). These prey taxa included stingarees (*Urolophus* sp.) (Fig. 2), eastern shovelnose rays (*Aptychotrema rostrata*), thornback cowfish (*Lactoria fornasini*), eastern smooth boxfish (*Anoplocapros inermis*), threebar porcupinefish (*Dicotylichthys punctulatus*), reef ocean perch (*Helicolenus percoides*) and a whiting (*Sillago* sp.). Notably, one *S. mokarran* stomach contained nine whole *Urolophus* sp., with a total stomach weight of 6.26 kg, and a second *S. mokarran* stomach contained three whole *Urolophus* sp. and three whole *A. rostrata* (6.15 kg) (Fig. 2; Table 1). In contrast, none of the *C. limbatus* stomachs contained recognisable prey, and maximum stomach weights were generally much lighter (range 0.19–0.71 kg) than those of *S. mokarran* (0.38–6.26 kg) (Table 1).

### Metabarcoding assays

After quality filtering, 1,264,497 Mifish (12S) reads, 1,157,551 Crust16Sshort reads, and 237,745 Uni18S reads were retained for analyses (Supplementary Table 1). The PCR negative controls showed low levels of human contamination for the 18S assay only (Table 1), whereas the control water sample collected alongside the *C. limbatus* stomach samples demonstrated contamination from Carcharhinidae DNA (most likely from *C. limbatus*). Carcharhinidae and/or Chondrichthyes reads were ubiquitous across all three genetic assays, including the *Urolophus* sp. and *A. rostrata* rays removed from the *S. mokarran* stomachs, and most likely represent the host predator’s DNA.

Taxa identified in the *S. mokarran* metabarcoding assays included crustaceans, cartilaginous fish and teleosts. Crustaceans and teleosts were identified in the *C. limbatus* stomach contents (Table 1). In most cases, stomach-content analyses on the *Urolophus* sp. and *A. rostrata* removed from the *S. mokarran* stomachs displayed high taxon similarity to that of the apex predators’ stomachs. For example, a comparison of 4H (*S. mokarran*) and 4SR (*Urolophus* sp.) revealed *Gnathophis* spp., *Lepidotrigla* spp., *Pseudorhombus* spp., and *Anoplocapros* spp. in common (Table 1).

### Testing the Russian-doll effect

**Figure 3:**
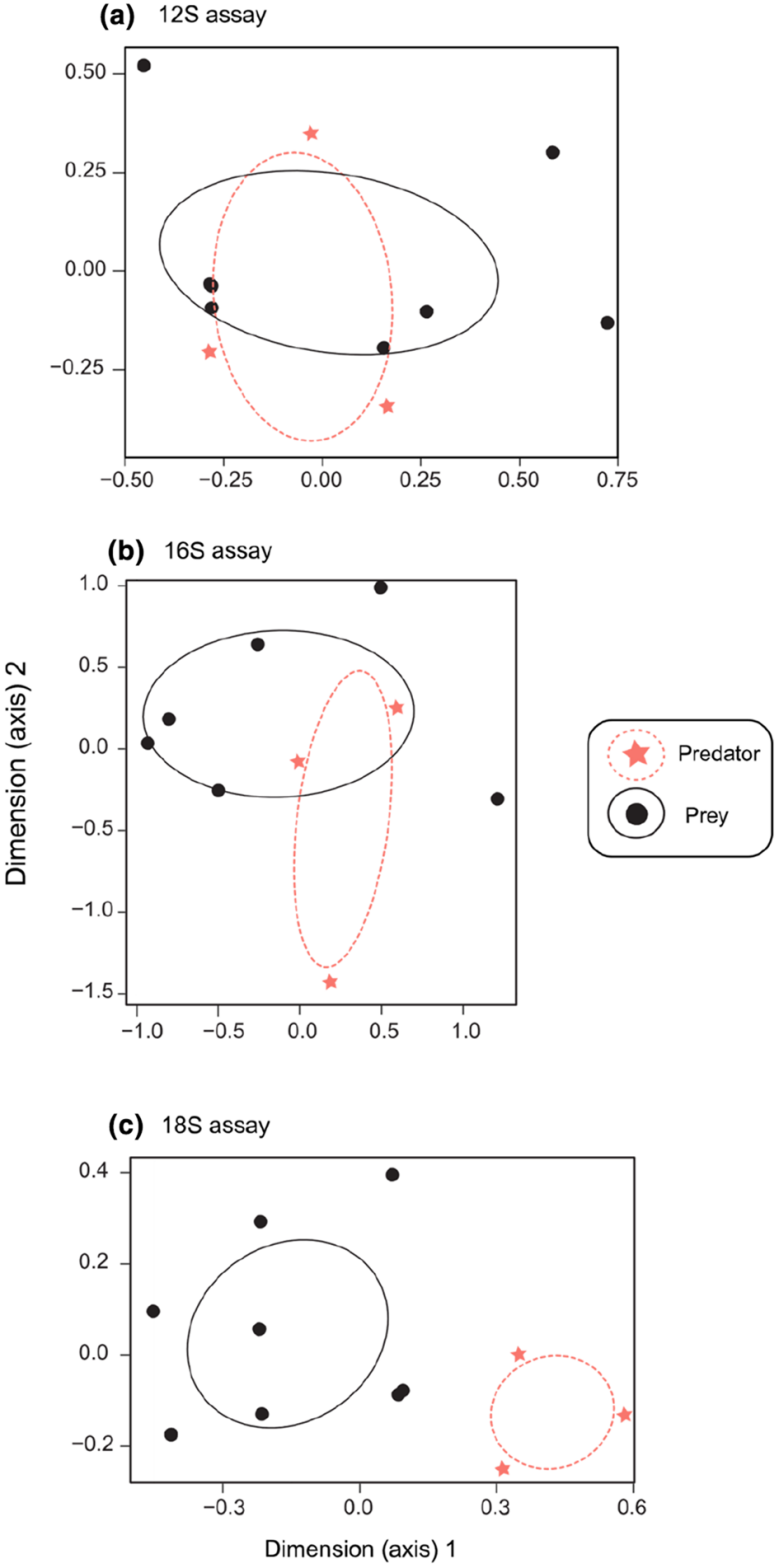
Kruskal’s non-metric multidimensional scaling (NMDS) plots of stomach-content metabarcoding assays for predators (*S. mokarran*; dotted lines and stars) vs. that of their whole prey (*Urolophus* sp. and *A. rostrata*; solid lines and circles), (**a**) 12S, (**b**) 16S, and (**c**) 18S assays, based on taxon and number of reads. For comparison, NMDS with stable solution from random starts showed the same pattern (not presented). Predator and prey comparisons based on taxon presence/absence only showed no significant differences for all three assays (not presented) (*p* > 0.05).

**Figure 4:**
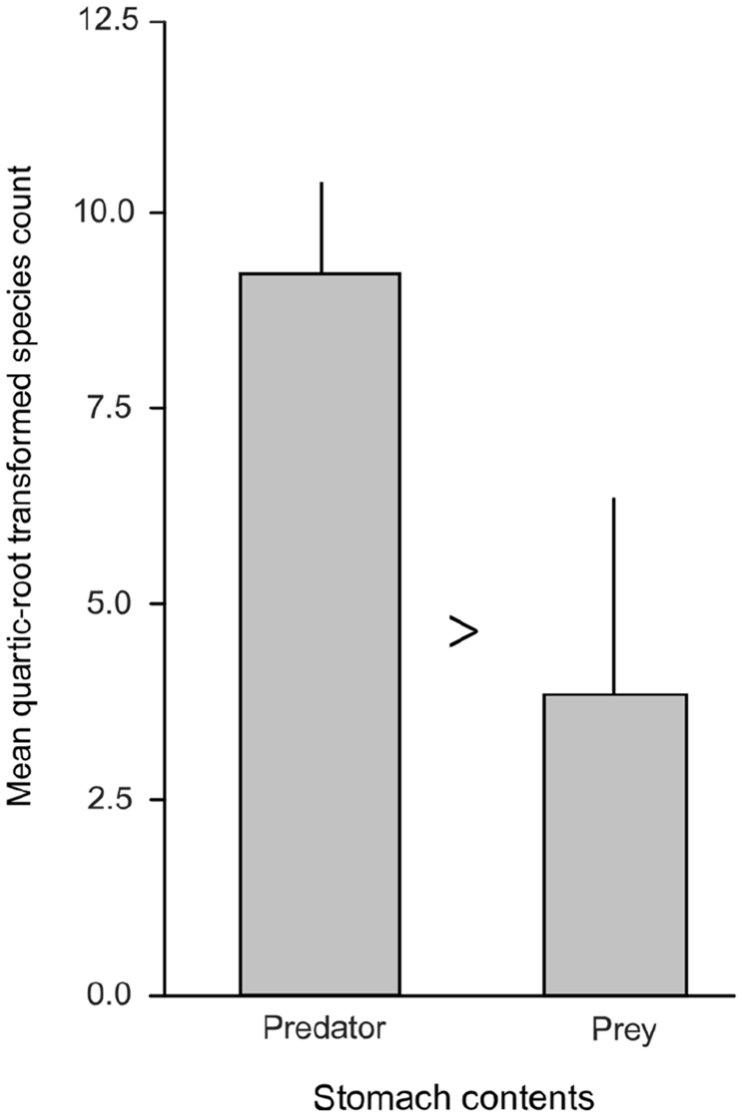
Mean (+/SE) number of reads for taxon ‘Carcharhinidae’, showing a statistically significant increase (marked as ‘ > ’, *p* < 0.05) of the number of reads from the predators’ stomach contents compared to that of their whole prey, likely reflecting elevated sequencing of host DNA for this assay (Chondricthyes and Actinopteri not presented).

Both of the NMDS tests and PERMANOVA showed no significant differences between stomach contents from predator and prey for the 12S and 16S metabarcoding assays (*p* > 0.05) for number of reads, and for presence/absence comparisons, but did show a significant difference for number of reads for the 18S assay only (*p* < 0.05, Fig. 3, Table 2). The SIMPER analysis showed that the dissimilarities between number of reads for 18S predator and prey comparisons were driven predominantly by Chondrichthyes (38%) and Carcharhinidae (30%) (Fig. 4), which were elevated in the predator, and Actinopteri (4%), which was elevated in the prey. These results indicate that taxonomic composition, and even the number of metabarcoding reads from stomach contents of predator and their whole prey, were not statistically different from one another, at least for 12S and 16S. Differences in the number of reads, but not taxonomic composition, for the 18S assay likely reflects increased host DNA sequenced for this marker (i.e. Chondrichthyes, Carcharhinidae; but not Actinopteri) (Fig. 4). One caveat of these analyses is that the sample size was small, with three replicates for *S. mokarran*.

**Table 2:** PERMANOVA results for three metabarcoding assays (12S, 16S, 18S) comparing the number of reads of taxa in stomach contents from predators (*S. mokarran*) vs. that of their whole prey (*Urolophus* sp. and *A. rostrata*).

Metabarcode	Df	F	*P*-value	Sig	*r*^2^
12S	1	0.50	0.87	Ns	0.05
16S	1	0.70	0.73	Ns	0.09
18S	1	7.88	0.01	Sig	4.47

## Discussion

The data collected here represent the first efforts at metabarcoding the stomach contents of *S. mokarran* and *C. limbatus* off eastern Australia. Our results support the general trends in the literature describing the diet of *S. mokarran* as being dominated by rays^14–16^, but also including teleosts and other sharks^12,13,17,18^, and reports of *C. limbatus* predominantly feeding on teleosts^20–22^. By ‘Russian-dolling’^39^ the stomach contents of these marine predators and their prey, we have also quantified some of the issues encountered when attempting to reconstruct trophic interactions from metabarcoding stomach contents.

Of the two species, *S. mokarran* dietary preferences were the most discernible, and clearly some had very recently fed in the general area (considering the regional abundances of some whole prey items)*.* Based on whole prey items, *S. mokarran* consumed stingrays and skates (Myliobatiformes and Rajiformes), but also teleosts including both slow- (*Anoplocapros inermis*) and fast-moving species (*Sillago* sp.). Observations of prey handling by *S. mokarran* are limited, however the species has been observed to use its laterally expanded head (cephalofoil) to immobilise prey on the ocean floor during both the pursuit of bottom-dwelling rays (southern stingray *Dasyatis americana*)^16^, and the post-capture manipulation of pelagic rays (spotted eagle ray *Aetobatus narinari*)^14^. Most of the prey items of the *S. mokarran* individuals identified here were bottom dwellers (e.g. rays: *Urolophus* sp., *A. rostrata* and perch: *H. percoides*), which supports the above mechanisms of prey-capture and -manipulation (e.g.^49–51^).

Unlike for *S. mokarran*, we found no whole fish in the digestive-tracts of the four *C. limbatus*. Nevertheless, metabarcoding indicated the presence of large species including *Platycephalus* spp. Several platycephalids occur nearshore off northern NSW, especially eastern bluespotted flathead (*P. caeruleopunctatus* up to 0.6 m TL) and, to a lesser extent, dusky flathead (*P. fuscus* up to 1.2 m TL)*.* Large individuals of these species were unlikely to be prey of other animals in the stomachs of *C. limbatus*, but in the absence of whole specimens any conclusions based on the exact platycephalid species are speculative.

Other primary target species identified by metabarcoding in both *S. mokarran* and *C. limbatus* included *Dexillus* spp., probably tufted sole *Dexillus muelleri*; the only known species from this genus, occurring off more tropical areas of Australia. This might imply these sampled sharks (5H, 6H, 1BT, and 4BT) moved southwards from a northern more, tropical foraging area^13^, or less likely that an as yet unidentifed species of *Dexillus* occurs in northern NSW waters. Five of the *S. mokarran* and two of the *C. limbatus* also had *Anoplocapros* spp. metabarcoding reads sequenced from their stomach contents, and we observed *A. inermis* scales in the stomach of one *S. mokarran*, suggesting some dietary overlap with *C. limbatus*. Indeed, *A. inermis* is the only species in this genus (of three total species, including *A. amygdaloides* and *A. lenticularis*) found off eastern Australia.

While some larger primary prey items of *S. mokarran* and to a lesser extent *C. limbatus* could be identified, it was difficult to ascribe predation sources to the smaller taxa, such as the sandy sprat *Hyperlophus vittatus* or biddies *Gerres* sp. (likely silver biddy *G. subfasciatus*, although this species overlaps with at least two other congeners in northern NSW) detected in the 12S assay. These species could either be primary prey of the sharks or secondary prey consumed by the platycephalids. The Russian-doll effect^39^ is exemplified well by comparing predator–prey stomach content metabarcoding reads. For those *S. mokarran* that contained whole prey items (e.g. Myliobatiformes), DNA metabarcoding assays yielded close matches between predator and prey stomach contents. It is therefore difficult to conclude whether the *S. mokkaran* (less likely) or *Urolophus* sp. or *Aptychotrema rostrata* (most likely) were feeding on smaller prey items, such as decapod crustaceans and fish species. We suggest that the chyme of both predator and (whole) prey intermixed with the other.

Similarly, our metabarcoding approach could not determine whether smaller sharks had been consumed by *S. mokarran* (as expected from the literature), due to the fact that all *S. mokarran* and their whole prey stomach samples included an abundance of 12S Carcharhinidae reads. However, the 16S assay did suggest these reads likely reflected the host predator’s DNA, considering *S. mokarran* reads were identified both from *S. mokarran* and *Urolophus* sp. stomach contents, but at very low abundances. Blocking primers designed to exclude the host predator’s DNA would be a worthwhile inclusion in future metabarcoding dietary studies (e.g.^36,52,53^), while shark-specific primers could also prove more informative on this topic^30^. Alternatively, increasing the depth of sequencing may facilitate detecting the low template (prey) fraction of the metabarcoding chyme despite the presence of host DNA that would otherwise swamp the PCR amplification process.

In this study, metabarcoding assays provided some insights into the dietary preferences of *S. mokarran* and *C. limbatus* off eastern Australia. These species appear to have some dietary overlap, but with consistency among prey species identified in studies of their feeding ecology from across their broader ranges^12–18,20–22^. *Sphyrna mokkaran* fed predominantly on Myliobatiformes and Rajiformes, but also teleosts, whereas *C. limbatus* fed predominantly on teleosts, which is consistent with its smaller body size and lack of cephalofoil that allows specialized feeding of benthic prey.

The Russian-doll effect made the reconstruction of trophic interactions from stomach metabarcoding data problematic. Extensive intermixing in situ between predator–prey digestive tracts was evident, which also limited the ability to discriminate between primary and secondary predators of smaller teleosts and crustacean taxa. The literature on the Russian-doll effect^39^ in metabarcoding dietary studies is limited (reviewed in^27,38^) but certainly deserves further attention (e.g.^54^). We suggest that while the approach offers some utility in identifying unseen taxa, confirmation via visual identification and across sufficient replication (ideally from various sampling methods) is still required to comprehensively understand primary dietary preferences.

## Supplementary Information

Supplementary Information.

## Data Availability

The datasets generated and analysed during the current study have been archived in Dryad: https://doi.org/10.5061/dryad.kd51c5b4s.
