# Supplementary material for: Secondary predation constrains DNA-based diet reconstruction in two threatened shark species
Source: Sci Rep. 2021 Sep 15;11:18350. doi: 10.1038/s41598-021-96856-w (PMC8443726; doi:10.1038/s41598-021-96856-w)
Supplement: Supplementary file 1 — Supplementary Information. [file 41598_2021_96856_MOESM1_ESM.docx]

**Table S1**. **18S, 16S, and 12S sequence information per stomach content sample collected from northern New South Wales, Australia**. Zero reads indicate no amplification, whereas “n/a” indicates that the sample was not processed. Minimum phred score for quality filtering (QF) was set to 30. Minimum length for QF was set to 250 base pairs (bp) for 18S, 80 bp for 16S, and 150 bp for 12S.

| **Sample ID** | **Species** | **Location** | **Date** | **Sex** | **Total length (mm)** | **18S post QF reads** | **18S unique reads** | **16S post QF reads** | **16S unique reads** | **12S post QF reads** | **12S unique reads** |
| --- | --- | --- | --- | --- | --- | --- | --- | --- | --- | --- | --- |
| 1H | *Sphyrna mokarran* | Lennox Head Beach, Lennox Head | 7/2/2018 | M | 2870 | 15773 | 8702 | 142214 | 6789 | n/a | n/a |
| 2H | *Sphyrna mokarran* | Lighthouse Beach, Ballina | 19/3/2018 | F | 3290 | 4663 | 1517 | 19 | 8 | n/a | n/a |
| 3H | *Sphyrna mokarran* | Shelly Beach, Ballina | 2/5/2018 | F | 3322 | 16765 | 7060 | 438 | 93 | 89924 | 6992 |
| 4H | *Sphyrna mokarran* | Main Beach, Evans Head | 17/2/2018 | F | 3080 | 12900 | 7400 | 108059 | 7106 | 73059 | 5180 |
| 5H | *Sphyrna mokarran* | Main Beach, Evans Head | 19/3/2018 | M | 2268 | 12248 | 8856 | 188016 | 9363 | 120532 | 6420 |
| 6H | *Sphyrna mokarran* | Main Beach, Evans Head | 19/3/2018 | M | 2850 | 6600 | 2202 | 103 | 36 | 65290 | 4057 |
| 7H | *Sphyrna mokarran* | Main Beach, Evans Head | 13/4/2018 | M | 2961 | 12924 | 5809 | 129257 | 5732 | 104915 | 8360 |
| CH | Control - *Sphyrna mokarran* | n/a | n/a | n/a | n/a | 0 | 0 | 0 | 0 | 0 | 0 |
| PCRneg1 | PCR negative 1 | n/a | n/a | n/a | n/a | 8126 | 2464 | 0 | 0 | 0 | 0 |
| 1BT | *Carcharhinus limbatus* | Shelly Beach, Ballina | 10/03/2018 | M | 1696 | 2322 | 1154 | 16 | 4 | 89912 | 8689 |
| 2BT | *Carcharhinus limbatus* | Shelly Beach, Ballina | 10/03/2018 | F | 2314 | 5240 | 1466 | 403 | 62 | 68992 | 5203 |
| 3BT | *Carcharhinus limbatus* | Shelly Beach, Ballina | 10/03/2018 | M | 1178 | 6828 | 3141 | 0 | 0 | 62342 | 5888 |
| 4BT | *Carcharhinus limbatus* | Shelly Beach, Ballina | 12/03/2018 | F | 2460 | 4020 | 1363 | 105 | 22 | 33110 | 2725 |
| CBT | Control - *Carcharhinus limbatus* | n/a | n/a | n/a | n/a | 7930 | 2613 | 0 | 0 | 1145 | 194 |
| PCRneg2 | PCR negative 2 | n/a | n/a | n/a | n/a | 1679 | 719 | 0 | 0 | 0 | 0 |
| 3A | *Urolophus* sp. | in gut of 3H | n/a | n/a | n/a | 9490 | 2129 | 5 | 3 | 60184 | 4526 |
| 3B | *Urolophus* sp. | in gut of 3H | n/a | n/a | n/a | 8149 | 3373 | 151315 | 3654 | 101188 | 6894 |
| 3C | *Urolophus* sp. | in gut of 3H | n/a | n/a | n/a | 6966 | 2587 | 66122 | 2815 | 12858 | 1418 |
| 3D | *Urolophus* sp. | in gut of 3H | n/a | n/a | n/a | 14465 | 3291 | 203 | 74 | 40663 | 2303 |
| 4SR | *Urolophus* sp. | in gut of 4H | n/a | n/a | n/a | 13037 | 6368 | 82197 | 3477 | 76959 | 5730 |
| 4SN | *Aptychotrema rostrata* | in gut of 4H | n/a | n/a | n/a | 16744 | 4175 | 86480 | 7824 | 76915 | 4811 |
| 4SN2 | *Aptychotrema rostrata* | in gut of 4H | n/a | n/a | n/a | 19464 | 9258 | 0 | 0 | 72864 | 2799 |
| 4 | *Urolophus* sp. | in gut of 4H | n/a | n/a | n/a | 13449 | 4438 | 96820 | 6830 | 53382 | 2598 |
| 7SR | *Urolophus* sp. | in gut of 7H | n/a | n/a | n/a | 17963 | 9260 | 105779 | 4661 | 60263 | 5319 |
